# Drones Do Not Drift between Nests in a Wild Population of *Apis cerana*

**DOI:** 10.3390/insects14040323

**Published:** 2023-03-27

**Authors:** Thomas Hagan, Julianne Lim, Rosalyn Gloag

**Affiliations:** Behaviour, Ecology and Evolution Lab, School of Life and Environmental Sciences, University of Sydney, Sydney, NSW 2006, Australia

**Keywords:** *Apis*, male dispersal, worker reproduction, sociality, invasive species

## Abstract

**Simple Summary:**

In honey bees, drones disperse to specific locations in order to mate before returning to their natal nests. However, in apiaries of *Apis mellifera*, drones have been witnessed returning to non-natal nests; this behavior may also sometimes occur at natural nests. This behavior could be due to the unnaturally high density of nests in apiaries. In this study, we genotyped colonies of the *Apis cerana* honey bee collected in its invasive range of Far North Queensland, Australia, to determine whether there is evidence of drone drift between colonies. We found no such evidence of drone drift. Some drones that do not match the resident queen’s genotype are instead likely due to queen turnover or worker reproduction.

**Abstract:**

The modes through which individuals disperse prior to reproduction has important consequences for gene flow in populations. In honey bees (*Apis* sp.), drones (males) reproduce within a short flight range of their natal nest, leaving and returning each afternoon within a narrow mating window. Drones are assumed to return to their natal nests as they depend on workers to feed them. However, in apiaries, drones are reported to regularly make navigation errors and return to a non-natal nest, where they are accepted and fed by unrelated workers. If such a “drone drift” occurred in wild populations, it could facilitate some further degree of dispersal for males, particularly if drones drift into host nests some distance away from their natal nest. Here, we investigated whether drone drift occurs in an invasive population of the Asian honey bee (*Apis cerana*). Based on the genotypes of 1462 drones from 19 colonies, we found only a single drone that could be considered a candidate drifter (~0.07%). In three other colonies, drones whose genotypes differed from the inferred queen were best explained by recent queen turnover or worker-laying. We concluded that drone drift in this population is low at best, and *A. cerana* drones either rarely make navigation errors in wild populations or are not accepted into foreign nests when they do so. We therefore confirm that drone dispersal distance is limited to the distance of daily drone flights from natal nests, a key assumption of both colony density estimates based on sampling of drone congregation areas and population genetic models of gene flow in honey bees.

## 1. Introduction

In hymenopteran social insects (ants, some bees, and wasps), dispersal often has a critical role in gene flow and inbreeding avoidance. Inbreeding in these species is particularly costly due to the method of sex determination typical for these species, Complementary Sex Determination (CSD) [1]. Under this system, sex is determined via zygosity at one or a few sex loci, with all haploid embryos developing as drones (males). Diploid embryos develop as females (workers or queens), provided that they are heterozygous for at least one sex locus. However, diploid embryos that are homozygous for all sex loci develop as “diploid males”, which are typically inviable, cannibalized as offspring, infertile, or produce infertile triploid offspring [1,2,3,4,5,6]. Therefore, modes of dispersal biased towards outbreeding will increase heterozygosity at sex loci and reduce rates of costly diploid male production in populations of social hymenoptera. This is especially relevant for small invasive or endangered hymenopteran populations [7], where genetic diversity is already low and the risks of inbreeding depression are highest [8,9,10].

In honey bees (genus: *Apis*), gene flow is principally determined by the movements of queens and drones. Movement of both sexes can occur prior to mating while post-mating movement occurs for queens only, as drones die during mating [11]. In order to mate, drones and virgin queens travel to common locations in the environment called drone congregation areas (DCAs) [12]. Drones may aggregate at DCAs in large numbers, up to the thousands, and these aggregations typically represent dozens of local colonies [13,14]. Until such a time that drones are ready to undertake a mating flight, they remain in their natal nest, being cared for by their sisters [15]. Therefore, before mating has occurred, dispersal is theoretically limited to the flight range of drones and queens around their natal nests, a range that may vary with life-history traits such as age [16]. After mating, however, dispersal may also occur through the movement of queens in reproductive swarms. Reproductive swarms are produced from parent colonies through fission and consist of a single, mated queen with a portion of the previous colony’s worker force [17,18]. These reproductive swarms establish a new nest away from the parent colony, at distances from as little as a few hundred meters [18] to up to 10 km [19]. The first queen that leaves in a reproductive swarm is the queen of the original colony, while the last virgin queen to emerge may either stay to inherit the old nest, or swarm herself so that the old nest is abandoned. Even if the queen in a swarm perishes during the swarms’ move, or if the last virgin queen perishes during her mating flight, the remaining workers may activate their ovaries, build nests, and begin laying unfertilized (drone) eggs [20,21,22].

The movement of drones between nests could passively increase flight ranges and impact gene flow. That is, drones could expand their effective flight range and mate at greater distances from their natal nest if they sometimes return to non-natal nests upon unsuccessful mating flights. This behavior is known as “drifting” [23,24,25,26]. Such drone drift occurs regularly in *Apis mellifera* hives kept in managed environments [24,26], where workers may allow males to enter the nest irrespective of whether it is the drones’ nest of origin. In these conditions, up to 89% of males may be non-natal [24,25], although drones often remain faithful to their natal colony [27]. Drone drift is assumed to occur when drones make recognition errors, mistaking other nearby managed nests (hives) for their natal nest. Corroborating this, some studies detailing drone drift have found associated directional effects [26], such as what would occur if drones used the relative position of the sun to determine the path home to their natal nest. Yet, whether drone drift occurs in wild honey bee populations is uncertain. In wild populations, rates of drone drift are presumably dependent on the navigational cues that drones use when travelling to and from DCAs, as well as the average proximity of nests to each other in space. While little is definitively known about how drones navigate to and from DCAs, they have been suggested to navigate local areas via the use of natural landmarks [28,29]. If drones are prone to errors in recognizing landmarks, and/or nests are located close to one another, then drones might be expected to sometimes drift between wild nests. Alternatively, some drones could navigate to and from DCAs by following their brothers. This may result in drones drifting into non-natal nests upon returning from mating flights if they follow non-sibling males. In fact, drones will readily follow other males during flight once congregated at DCAs [11], but whether they respond to such cues when returning back to nests is unknown. Regardless of cause, significant rates of drone drift between nests (especially non-adjacent nests) could result in increased male dispersal. Such movement would have important implications for our understanding of gene flow and the role that male dispersal plays in preventing inbreeding in honey bee mating systems.

In this study, we investigated the rate of inter-nest drift by drones in Australia’s invasive population of the Asian honey bee (*Apis cerana*). *A. cerana* is native to much of Asia and has become invasive in several Pacific regions east of its natural range in recent decades [30,31]. First detected in Cairns, Australia, in 2007 [30], the population is now established throughout a local region of approximately 10,200 km^2^ and contains at least 10,000 colonies [32]. This population has significantly reduced genetic diversity relative to native-range populations [33] and was likely founded by a single colony [34]. Due to the reduction in genetic diversity at the sex locus, this population has significantly higher levels of infertile diploid male offspring compared with native range populations (14% per colony compared to <1% per colony [32,33]. Therefore, drone drift could be particularly impactful in reducing inbreeding risk for this population. Specifically, we asked: (i) do queenright colonies contain adult drones that are not sons of the queen?, and (ii) are these drones likely to be the product of drift from other nests?

## 2. Materials and Methods

*Colony Collection*

To assess rates of drone drift, we genotyped 1376 adult drones (mean drones per colony = 77, range 34–189) from colonies collected between 2016 and 2021 (N = 19). We chose colonies where we had collected at least 20 adult drones from the colony to be included in this study. We deemed all these colonies to be queen right prior to inclusion in the study, based on the presence of worker brood. We located colonies via reports from the public to Queensland Biosecurity, local beekeepers, or to us directly. These colonies were collected from throughout the invasive range, although most were collected in the suburbs of Cairns, Far North Queensland. Once collected, colonies (either swarms or nests with comb) were cooled below 0 °C to euthanize them. We were not aware of other nests close in proximity to the colonies included in this study, although they may have been present. Over the course of our work in this population, we have observed occasional instances of two nests located within a few meters of each other.

*Genotyping*

We extracted DNA from one hind leg of each drone and eight workers per colony using the Chelex protocol [35]. We then genotyped all the bees at eight unlinked microsatellite loci (A107, Ac1, Ac3, Ac26, Ac27, Ac32, Ac35, & B124 [36,37]) and at a polymorphic fragment of the hypervariable region of the sex locus (*csd*) using standard PCR conditions (94 °C for 5 min, 35 cycles of 94 °C for 30 s, 56 °C for 30 s, and 72 °C for 30 s, with a final extension at 72 °C for 15 min). We amplified DNA in 5-µL reactions (1 × reaction buffer, 2.5 mM MgCl_2_, 0.16 mM dNTP mixture, 0.32–0.48 µM of fluorescent dye-labelled primers, and their unlabeled pair, Applied Biosystems, Waltham, MA, USA and Macrogen, Seoul, Republic of Korea, 0.2 units of Taq polymerase and 1 µL of extracted DNA). Primer pairs for *csd* are described in Gloag et al. 2017. In this approach, *csd* is amplified using a population-specific set of three primer pairs. These primers allow for the identification of the seven *csd* alleles present in Australia’s *A. cerana* population based on differences in allele length. In *A. cerana*, *csd* zygosity determines sex, where hemizygous individuals develop as males, heterozygous individuals develop as females, and homozygous individuals are inviable as they are cannibalized before developing as infertile diploid males. The *csd* locus is useful in determining maternity as offspring must share at least one *csd* allele with that of its mother, whom is definitively heterozygous, and the locus has higher allelic diversity than other regions of the genome in this invasive population (7 alleles [32]). The chosen microsatellite loci have been previously shown to have maintained some polymorphism in this population (2–4 alleles per locus) and are therefore also useful in determining maternity. PCR products were electrophoresed on a 3130xl Genetic Analyzer and allele calling was performed using GeneMapper 5 (Applied Biosystems, Waltham, MA, USA).

*Maternity Identification*

We used the genotypes of eight workers to determine the alleles of the natal queen for each colony. Amplification failure rates per locus were low at approximately 8% (range: 4–18%), meaning that queen genotypes were easily assessable. As workers must share an allele with the queen, queen genotypes were identified by finding the two alleles among workers so that each worker carried at least one of them per locus. If all workers were homozygous at a locus, we assumed that the queen was likewise homozygous, given that the chance of a heterozygous queen producing 6–8 offspring that shared only one of her alleles at a locus is ~0.4–1.6%. In a few cases, after determining the queen’s genotype from the most common alleles, we identified a worker that did not share an allele with the queen (this was the case for one worker in each of the two colonies). We assumed that these two workers were non-natal and excluded them from the determination of the queen’s genotype. Worker drift is known to occur at low rates in *A. cerana* and some other Asian honey bee species [38,39].

After determining the genotype of the queen in each colony, we looked for evidence of drone genotypes inconsistent with being the queen’s sons. Drones are produced via arrhenotoky (i.e., from unfertilized eggs) and all sons of the colony’s queen will therefore carry one of her two alleles at every locus. If a drone did not share one allele with the queen at each locus, we noted this drone as a “possible drifter”. However, there are two additional scenarios under which drones could be natal yet still have genotypes that are inconsistent with our inferred queens. First, colonies that experienced a recent queen change may contain sons of both the current queen and the previous queen. In this case, we would expect all the “possible drifters” to be consistent with being sons of a single additional mother, and for the inferred genotypes of the two mothers to be consistent with being a mother-daughter pair (i.e., a daughter queen had inherited the colony from her mother a few weeks prior to our sampling). Second, colonies may contain some drones that are the sons of workers rather than queens. In *Apis* spp., workers are capable of activating their ovaries to lay unfertilized eggs that develop into haploid drones [34,40,41]. When the queen is present, such cases of worker-laid eggs are typically policed (detected and eaten) by other workers, and “cheating” workers only very occasionally evade detection (in *A. cerana* < 1% male brood is worker-laid [22]). However, when colonies become queenless and cannot rear a replacement queen, worker policing will cease and worker-laid eggs will be reared in large numbers [42]. Although we aimed to select only queenright colonies for our analysis, after genotyping, we suspected that one of our included colonies may actually have been queenless, due to the very large number of non-queen drones. To investigate this further, we also measured the head sizes of all drones included in this study, using handheld digital calipers (nearest 0.01 mm). This is because workers in queenless colonies will often lay and rear their eggs in worker cells (which are smaller) rather than drone cells, resulting in smaller-bodied drones [34,43]. In *A. cerana*, head widths under 3.2 mm are very likely worker-laid drones [34]. We therefore considered that colonies with a high proportion of drones in this size category were very likely to be colonies of laying workers. We therefore excluded any colonies in which queen turnover or worker-laying seemed likely from our analysis of drift.

## 3. Results

**Figure 1:**
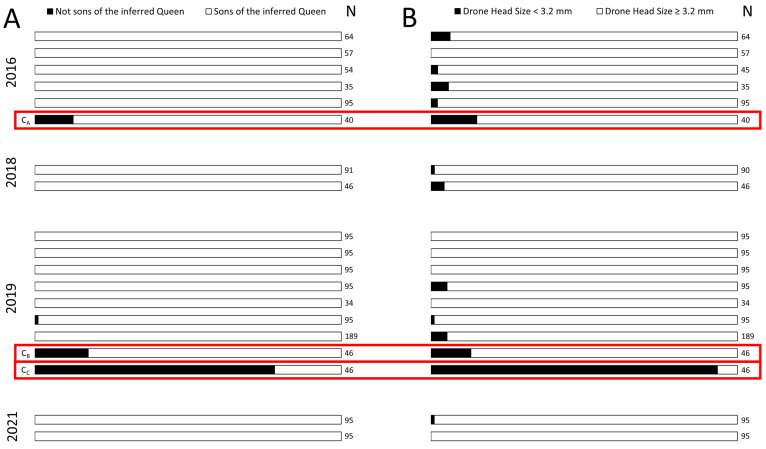
Each bar represents a colony used in this study, with the three colonies with high proportions of “non-queen” drones indicated in red and labelled as C_A_, C_B_, and C_C_. (**A**): The proportion of drones that do not match the genotype of the queen. In most colonies, all, or almost all, drones match the genotype of the queen, except for C_A_, C_B_, and C_C_ wherein 12.5%, 17.4%, and 78.3% of drones do not match the queen’s genotype. (**B**): The proportion of drones with very small head sizes (<3.2 mm) in each colony. Drones with head sizes in this category have typically been reared in worker-cells and not drone cells and therefore are indicative of colonies with laying workers.

We found low rates of “candidate drifter” drones in queenright wild nests of *Apis cerana* in Australia’s invasive population (i.e., drones with a genotype that were inconsistent with being sons of queens). All drones were queen-laid in 15 of our 19 study colonies (Figure 1A). Of the remaining colonies, one colony had a lone drone that was not the son of the queen, and three colonies were deemed to likely be cases of either recent queen turnover or worker-laying (Figure 1A).

**Figure 2:**
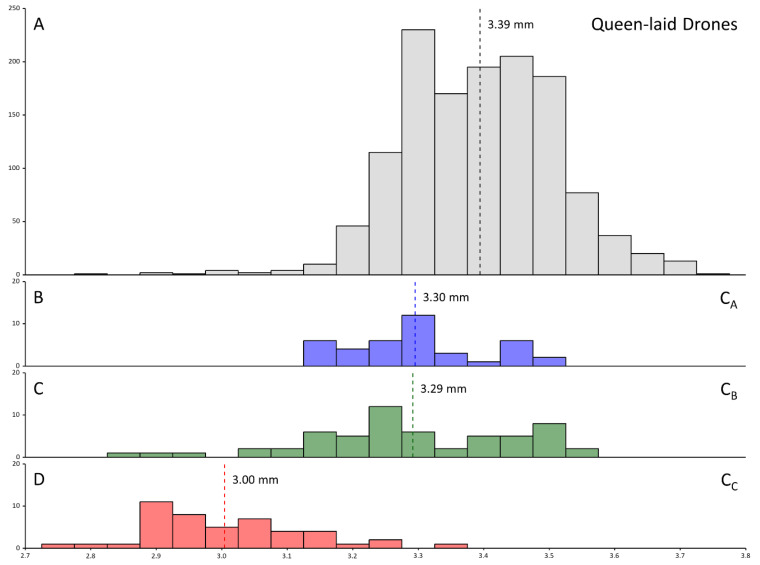
Histograms of the head width of drones used in this study. Dashed lines indicate the mean of each distribution. (**A**)—The head size distribution of all drones from queenright colonies in this study whose genotype indicated they were sons of the queen. (**B**,**C**)—The head size distribution of drones from colony C_A_ and C_B_, respectively. The mean head size is >3.2 mm, indicating that workers are not reproducing in this colony. (**D**)—The head size distribution of drones from colony C_C_. The mean head size is <3.2 mm, indicating that workers are reproducing in this colony.

Colonies C_A_ and C_B_ were deemed to be likely instances of recent queen change. Two of these colonies had moderate rates of drones that were not sons of the queen (C_A_ = 12.5%; N = 5/40 drones, and C_B_ = 17.4%; N = 8/46 drones), while in the final colony, most drones had genotypes inconsistent with being the queen’s sons (C_C_ = 78.3%; N = 36/46 drones). This is because all drones in these colonies that were inconsistent with being the offspring of the inferred queen (based on worker genotypes) were instead consistent with a single second mother, and the two inferred mothers per colony were consistent with a mother-daughter queen turnover. The size distribution of drones in these colonies was not suggestive of worker-laying (Figure 2B,C).

Meanwhile, Colony C_C_ was a very likely case of a colony with laying workers as it had high rates of drones with small head sizes (C_C_ = 93.5%; N = 43/46, Figure 2D) and a high diversity of drone genotypes (i.e., drones were the product of many mothers). Interestingly, C_C_ also had worker brood (ranging from eggs to larva to pupa), indicating the presence of a laying queen. Therefore, this colony appeared to be an aberrant case of workers laying drones, despite the queen’s presence (or possibly had had a period of queenlessness and worker-laying, prior to a new queen inheriting the colony).

## 4. Discussion

We found no evidence of significant levels of adult drones drifting between wild nests of *A. cerana*. We therefore conclude that either drones rarely make errors when orientating to and from nests in wild populations, or that when such navigation errors are made, the drones are repelled or rejected by workers in foreign nests. When colonies with likely cases of queen turnover events or high worker-laying were excluded, we found only one candidate drifter among our sampled queenright colonies (i.e., one drone with a genotype inconsistent with being a son of the queen; ~0.07% drone brood). Rather than being a drifter, this drone may well have also been born from a cheating worker, given that previously reported rates of laying workers in queenright colonies of *A. cerana* are sometimes as high as 1.1% of male pupae [22,44]. Most nests in our study were not known to be located within close proximity to other nests. It remains possible, therefore, that where nests are close to one another, drone drift may occur. However, if drone drift is limited only to such cases, then it would have little impact on estimates of drone dispersal ability. In all, we conclude that in wild populations of *A. cerana*, drifting behavior of drones is rare at best, and is unlikely to have significant impacts on either gene flow or colony-level genetic load.

High rates of drone drift have been reported in managed honey bee colonies, but this is likely an artefact of the high colony densities of these environments. Such drifting behavior is best documented in *A. mellifera*, where drones regularly return from drone flights to non-natal colonies within apiaries [25,26,45]. In these cases, drone drift is not random, but has a directional bias that corresponds to nest position (e.g., drones had a tendency to drift in a southward direction [26]) and decreases with greater distances between hives [23]. Both patterns suggest navigational errors on the part of drones. The lack of drifted drones in our study of wild nests suggests that drones are ordinarily adept at using visual and scent cues to navigate from DCAs to their natal nests and only become confused, thus returning to the wrong nest, under the artificially high colony density of apiaries. Alternatively, rates of drone drifting could be principally dictated by the behavior of workers at nest entrances. In apiary contexts, plentiful resources may reduce guarding by workers and increase the likelihood that foreign drones are accepted into the nest. This behavior may be absent in wild contexts where resources are scarcer. The rejection of non-natal drones by workers is expected if resources are limited, as colonies that tolerate high levels of drift from unrelated drones would likely incur fitness costs due to increased resource consumption and disease transfer. Such flexible thresholds for the tolerance of non-natals are well-documented in honey bees with respect to foreign workers entering nests [46,47]. Indeed, while workers sometimes make recognition errors of non-natal workers in *Apis* species generally [48,49], *Apis cerana* is particularly adept at rejecting non-natal drifting workers [39]. In all, a lower likelihood of drone navigation error and a higher chance of rejection by workers in combination probably reduces rates of drone drift in wild populations relative to apiary environments.

In our search for drifting drones, we identified two colonies with moderate proportions of drones inconsistent with being the offspring of a single queen (12–17%), and one colony with a high proportion of drones inconsistent with being the offspring of the queen (>70%), despite evidence of queen presence in all colonies (i.e., young to mature worker brood). In the former cases (colonies C_A_ and C_B_), genotypes of drones and workers suggested that these were very likely cases of queen turnover; that is, these colonies recently acquired new queens and offspring were therefore the progeny of two mothers (old and new queen). However, in the case of colony C_C_, we conclude that high rates of worker reproduction best explained the surprising genotypic diversity of drones in the colony, even though the colony had recent worker brood and therefore, should have a queen. Consistent with worker-laying described in previous studies [34,43], drones in this colony were smaller on average than typical queen-laid drones. During periods of queenlessness, *Apis* workers cease to police each other’s eggs and instead activate their ovaries to produce a final batch of unfertilized, haploid (male) offspring before the colony perishes [20,21,22]. Queenless colonies of laying workers use all available cells and, as most cells in a nest are worker-sized, the drones produced by laying workers are typically smaller than those that are laid by queens and are reared exclusively in larger drone cells [34,43]. This behavior is well documented in *A. cerana* and is predicted to have contributed to the maintenance of some genetic diversity in Australia’s invasive *A. cerana* after its founding bottleneck [34], as well as its now large population (approx. over 10,000 colonies; [32]). Furthermore, *Apis* workers may occasionally engage in active drifting behavior to “cheat”, that is, activate their ovaries and lay eggs in foreign nests [50,51]. Such behavior is rare and foreign workers are usually rejected, but may be accepted if the colony is queenless and thus further increase the genetic diversity of drones in colonies of laying workers [39]. We propose that, in the case of colony C_C_, workers were behaving as though the colony was in a queenless state because the resident queen was any of (1) young and unmated, (2) had ceased producing new worker brood (in preparation for swarming), or (3) had otherwise ceased to provide the typical cues used to regulate worker reproductive behavior [52]. Whether such worker-laying is common during certain transient phases of the *A. cerana* reproductive colony cycle, or whether this colony is an anomaly, is unclear and will require further study. Notably, however, the diversity of sex alleles (*csd* alleles) among the drones of colony C_C_ was high, with all seven of the populations’ *csd* alleles being represented at close to equal frequencies. It seems probable that a high incidence of worker-laid males is a more important driver of gene flow and inbreeding avoidance in *Apis* populations than any movement of adult drones between nests.

Social insect societies are dominated by females and the behaviors of their male counterparts are generally less documented. Yet, understanding the behaviors of males is critical to our ability to assess population density, model population dynamics, and accurately track gene flow in social insect populations. Where previous studies have documented the movement of drones between hives in apiaries, this is likely to be an artefact of the density and placement of colonies in those environments, as we found no evidence in this study of such drone movement between nests in a wild *A. cerana* population. We therefore confirm a key behavioral assumption within *Apis*: that drones captured at congregation areas originate from their nest of birth.

## Data Availability

The data presented in this study are openly available in FigShare at [https://doi.org/10.6084/m9.figshare.22186816.v1 (accessed on 23/03/2023)].
